# Both Galactosaminogalactan and α-1,3-Glucan Contribute to Aggregation of *Aspergillus oryzae* Hyphae in Liquid Culture

**DOI:** 10.3389/fmicb.2019.02090

**Published:** 2019-09-13

**Authors:** Ken Miyazawa, Akira Yoshimi, Motoaki Sano, Fuka Tabata, Asumi Sugahara, Shin Kasahara, Ami Koizumi, Shigekazu Yano, Tasuku Nakajima, Keietsu Abe

**Affiliations:** ^1^Laboratory of Applied Microbiology, Department of Microbial Biotechnology, Graduate School of Agricultural Science, Tohoku University, Sendai, Japan; ^2^ABE-Project, New Industry Creation Hatchery Center, Tohoku University, Sendai, Japan; ^3^Genome Biotechnology Laboratory, Kanazawa Institute of Technology, Hakusan, Japan; ^4^Department of Environmental Sciences, School of Food, Agricultural and Environmental Sciences, Miyagi University, Taiwa, Japan; ^5^Department of Biochemical Engineering, Graduate School of Engineering, Yamagata University, Yonezawa, Japan; ^6^Laboratory of Microbial Resources, Department of Microbial Biotechnology, Graduate School of Agricultural Science, Tohoku University, Sendai, Japan

**Keywords:** α-1,3-glucan, *Aspergillus oryzae*, cell wall, galactosaminogalactan, hyphal aggregation, recombinant-protein production

## Abstract

Filamentous fungi generally form aggregated hyphal pellets in liquid culture. We previously reported that α-1,3-glucan-deficient mutants of *Aspergillus nidulans* did not form hyphal pellets and their hyphae were fully dispersed, and we suggested that α-1,3-glucan functions in hyphal aggregation. However, *Aspergillus oryzae* α-1,3-glucan-deficient (AGΔ) mutants still form small pellets; therefore, we hypothesized that another factor responsible for forming hyphal pellets remains in these mutants. Here, we identified an extracellular matrix polysaccharide galactosaminogalactan (GAG) as such a factor. To produce a double mutant of *A. oryzae* (AG-GAGΔ), we disrupted the genes required for GAG biosynthesis in an AGΔ mutant. Hyphae of the double mutant were fully dispersed in liquid culture, suggesting that GAG is involved in hyphal aggregation in *A*. *oryzae*. Addition of partially purified GAG fraction to the hyphae of the AG-GAGΔ strain resulted in formation of mycelial pellets. Acetylation of the amino group in galactosamine of GAG weakened GAG aggregation, suggesting that hydrogen bond formation by this group is important for aggregation. Genome sequences suggest that α-1,3-glucan, GAG, or both are present in many filamentous fungi and thus may function in hyphal aggregation in these fungi. We also demonstrated that production of a recombinant polyesterase, CutL1, was higher in the AG-GAGΔ strain than in the wild-type and AGΔ strains. Thus, controlling hyphal aggregation factors of filamentous fungi may increase productivity in the fermentation industry.

## Introduction

The hyphae of filamentous fungi generally form aggregated pellets in liquid culture. Although filamentous fungi have been used for industrial production of enzymes and secondary metabolites for a long time (Abe et al., 2006; Kobayashi et al., 2007), hyphal pellet formation decreases productivity in liquid culture (Driouch et al., 2010; Karahalil et al., 2017). Formation of hyphal pellets might be related to a property of the cell surface (Beauvais et al., 2014), and elucidation of the relationship between hyphal aggregation and cell surface components, especially polysaccharides, is needed.

The fungal cell wall is essential for survival because it maintains the cell’s shape, prevents cell lysis, and protects cells from environmental stresses (Yoshimi et al., 2016). Fungal cell walls are composed mainly of polysaccharides. In *Aspergillus* species, the cell wall is composed of α-glucan (mainly α-1,3-glucan), β-1,3/1,6-glucan, galactomannan, and chitin (Latgé, 2010; Yoshimi et al., 2016, 2017). Cell walls of some filamentous fungi are covered with extracellular matrix, which is composed mainly of polysaccharides, including α-glucan (α-1,3-glucan with a small amount of α-1,4-linkage), galactomannan, or galactosaminogalactan (GAG) (Lee and Sheppard, 2016; Sheppard and Howell, 2016).

We reported that the Δ*agsB* and Δ*agsA*Δ*agsB* strains of *Aspergillus nidulans* have no α-1,3-glucan in the cell wall (Yoshimi et al., 2013) and their hyphae are fully dispersed in liquid culture, whereas the wild-type strain forms aggregated pellets. In *Aspergillus fumigatus*, addition of α-1,3-glucanase prevents aggregation of germinating conidia (Fontaine et al., 2010). These findings strongly suggest that α-1,3-glucan is an adhesive factor. We disrupted the three α-1,3-glucan synthase genes in the industrial fungus *Aspergillus oryzae* (Δ*agsA*Δ*agsB*Δ*agsC*; AGΔ) and confirmed the loss of α-1,3-glucan in the cell wall of the AGΔ strain, but the strain still formed small hyphal pellets in liquid culture (Miyazawa et al., 2016). Although the AGΔ hyphae were not fully dispersed, the strain produced more recombinant polyesterase (cutinase) CutL1 than did a wild-type strain (WT-cutL1) because of the smaller pellets of the AGΔ strain (Miyazawa et al., 2016). We predicted that another cell wall or cell surface component is responsible for hyphal aggregation in the AGΔ strain. Identification of this factor, distinct from α-1,3-glucan, is important, because full dispersion of *A. oryzae* hyphae would enable higher cell density and increase production of commercially valuable products in liquid culture.

GAG is a hetero-polysaccharide composed of linear α-1,4-linked galactose (Gal), *N*-acetylgalactosamine (GalNAc), and galactosamine (GalN). GAG is an important pathogenetic factor in the human pathogen *A. fumigatus* (Fontaine et al., 2011; Lee et al., 2015); it is involved in adherence to host cells, biofilm formation, and avoidance of immune response by masking β-1,3-glucan and chitin (Gravelat et al., 2013; Sheppard and Howell, 2016). Disruption of genes encoding the transcription factors StuA and MedA significantly decreases GAG content and has led to identification of the *uge3* (UDP-glucose 4-epimerase) gene (Gravelat et al., 2013). Four genes (*sph3*, *gtb3*, *ega3*, and *agd3*) located near *uge3* have been identified (Lee et al., 2016). In *stuA* and *medA* gene disruptants, these five genes are downregulated, suggesting that they are co-regulated by StuA and MedA (Lee et al., 2016). GAG biosynthesis by the five encoded proteins is predicted in *A. fumigatus* (Bamford et al., 2015; Sheppard and Howell, 2016). First, the epimerase Uge3 produces UDP-galactopyranose (Gal*p*) from UDP-glucose and UDP-*N*-GalNAc from UDP-*N*-acetylglucosamine (GlcNAc) (Gravelat et al., 2013; Lee et al., 2014). Second, glycosyltransferase Gtb3 seems to polymerize UDP-Gal*p* and UDP-GalNAc and export the polymer from the cytoplasm (Speth et al., 2019), although Gtb3 has not yet been characterized. Third, deacetylase Agd3 deacetylates the synthesized GAG polymer (Lee et al., 2016). The predicted glycoside hydrolase Ega3 has yet to be characterized. Sph3 belongs to a novel glycoside hydrolase family, GH135, and is essential for GAG production (Bamford et al., 2015), but its role in GAG synthesis remains unknown.

Here, we confirmed that *A. oryzae* has the GAG biosynthetic gene cluster. We disrupted *sphZ* (ortholog of *A. fumigatus sph3*) and *ugeZ* (ortholog of *uge3*) in the wild-type and AGΔ strains to produce Δ*sphZ*Δ*ugeZ* (GAGΔ) and Δ*agsA*Δ*agsB*Δ*agsC*Δ*sphZ*Δ*ugeZ* (AG-GAGΔ), respectively. In liquid culture, the hyphae of the AG-GAGΔ strain were fully dispersed, suggesting that GAG plays a role in hyphal adhesion in *A. oryzae*, along with α-1,3-glucan. Using the wild-type, AGΔ, GAGΔ, and AG-GAGΔ strains of *A. oryzae*, we characterized hyphal aggregation and discuss its mechanism in *A. oryzae*. Our findings may have wide implications, because the genomes of many filamentous fungi encode enzymes required for α-1,3-glucan or GAG biosynthesis, or both (Lee et al., 2016; Yoshimi et al., 2017).

## Materials and Methods

### Strains and Growth Media

Strains used are listed in Table 1. *Aspergillus oryzae* NS4 (*sC*^−^, *niaD*^−^) with Δ*ligD* (Δ*ligD*::*sC*, Δ*adeA*::*ptrA*) was used for all genetic manipulations (Mizutani et al., 2008). All *A. oryzae* strains were cultured in standard Czapek-Dox (CD) medium as described previously (Yoshimi et al., 2013; Miyazawa et al., 2016). The *niaD*^−^ strains were cultured in CDE medium (CD medium containing 70 mM sodium hydrogen L(+)-glutamate monohydrate as the nitrogen source instead of sodium nitrate).

**Table 1:** Strains used in this study.

Strain	Genotype	Reference
Wild type	Δ*ligD*::*sC*, Δ*adeA*::*ptrA*, *niaD*^−^, *adeA*^+^	Mizutani et al., (2008)
Δ*agsA*Δ*agsB*Δ*agsC* (AGΔ)	Δ*ligD*::*sC*, Δ*adeA*::*ptrA*, *niaD*^−^, *adeA*^+^, *agsA*::*loxP*, *agsB*::*loxP*, *agsC*:: *loxP*	Miyazawa et al., (2016)
Δ*sphZ*Δ*ugeZ* (GAGΔ)	Δ*ligD*::*sC*, Δ*adeA*::*ptrA*, *niaD*^−^*, sphZugeZ*::*adeA*	This study
Δ*agsA*Δ*agsB*Δ*agsC*Δ*sphZ*Δ*ugeZ* (AG-GAGΔ)	Δ*ligD*::*sC*, Δ*adeA*::*ptrA*, *niaD*^−^, *agsA*::*loxP*, *agsB*::*loxP*, *agsC*::*loxP, sphZugeZ*::*adeA*	This study
WT-cutL1	Δ*ligD*::*sC*, Δ*adeA*::*ptrA*, *niaD*^−^, *adeA*^+^, P*glaA142*-*cutL1*::*niaD*	Miyazawa et al., (2016)
AGΔ-cutL1	Δ*ligD*::*sC*, Δ*adeA*::*ptrA*, *niaD*^−^, *adeA*^+^, *agsA*::*loxP*, *agsB*::*loxP*, *agsC*:: *loxP*, P*glaA142*-*cutL1*::*niaD*	Miyazawa et al., (2016)
AG-GAGΔ-cutL1	Δ*ligD*::*sC*, Δ*adeA*::*ptrA*, *niaD*^−^, *agsA*::*loxP*, *agsB*::*loxP*, *agsC*::*loxP, sphZugeZ*::*adeA*, P*glaA142*-*cutL1*::*niaD*	This study

Conidia of *A*. *oryzae* used to inoculate flask cultures were isolated from cultures grown on malt medium, as described previously (Miyazawa et al., 2016). YPD medium containing 2% peptone (Becton Dickinson and Company, Sparks, Nevada, USA), 1% yeast extract (Becton Dickinson and Company), and 2% glucose was used for flask culture to analyze growth characteristics. YPM medium containing 2% peptone, 1% yeast extract, and 2% maltose was used for flask culture to evaluate production of recombinant cutL1.

### Construction of Dual *sphZ ugeZ* Gene Disruptant in *Aspergillus oryzae*

The sequences of all primers are listed in Table 2. Fragments containing the 3′ non-coding regions of *ugeZ* (amplicon 1) and *sphZ* (amplicon 2) derived from *A. oryzae* genomic DNA, and the *adeA* gene (amplicon 3) from the TOPO-2.1-adeA plasmid (Miyazawa et al., 2016), were amplified by PCR. Amplicon 1 was amplified with the primers sphZ+ugeZ-LU and sphZ+ugeZ-LL+ade, amplicon 2 with the primers sphZ+ugeZ-RU+ade and sphZ+ugeZ-RL, and amplicon 3 with the primers sphZ+ugeZ-AU and sphZ+ugeZ-AL. The primers sphZ+ugeZ-LL+ade, sphZ+ugeZ-AU, and sphZ+ugeZ-AL were chimeric; each contained a reverse-complement sequence for PCR fusion. The PCR products were gel-purified and used as substrates for the second round of PCR with the primers sphZ+ugeZ-LU and sphZ+ugeZ-RL to fuse the three fragments (Supplementary Figure S1A). The resulting major PCR product was gel-purified and used to transform *A. oryzae* wild-type and AGΔ strains (Supplementary Figure S1B). Disruption of the *sphZ* and *ugeZ* genes was confirmed by Southern blot analysis (Supplementary Figure S1C).

**Table 2:** PCR primers used in this study.

Purpose	Primer name	Sequence (5′ to 3′)
*sphZ*, *ugeZ* disruption		
	sphZ+ugeZ-LU	TCTCCATAGTGTTCACCA
	sphZ+ugeZ-LL + Ade	ATATACCGTGACTTTTTAGCACAACATTGGAGCTACT
	sphZ+ugeZ-RU + Ade	AGTTTCGTCGAGATACTGCGCGTTGTCATATTTGCAAG
	sphZ+ugeZ-RL	AGGGCTCAGAATACGTATC
	sphZ+ugeZ-AU	AGTAGCTCCAATGTTGTGCTAAAAAGTCACGGTATATCATGAC
	sphZ+ugeZ-AL	TTGCAAATATGACAACGCGCAGTATCTCGACGAAACTACCTAA
Quantitative PCR		
	agsA-RT-F	CAAACCTGGAGAGACGCGAT
	agsA-RT-R	CGAGGGTATTCGCAAGTGTTG
	agsB-RT-F	GAACTTTGTCGCGGTCATCCTTCAG
	agsB-RT-R	CCAAGGGAGGTAGTAGCCAATG
	agsC-RT-F	TTGGAGACGGACCATCACTG
	agsC-RT-R	GTTGCAGGTCTCGTTGTACTC

### Analysis of Growth Characteristics of *Aspergillus oryzae* in Liquid Culture

Conidia (final concentration, 1 × 10^5^/ml) of the wild-type, AGΔ, GAGΔ, and AG-GAGΔ strains were inoculated into 50-ml of YPD medium in 200-mL Erlenmeyer flasks and rotated at 120 rpm at 30°C for 24 h. The mean diameter of the hyphal pellets was determined as described previously (Miyazawa et al., 2016).

### Scanning Electron Microscopy

Conidia (final concentration, 1 × 10^5^/ml) of the wild-type, AGΔ, GAGΔ, and AG-GAGΔ *A. oryzae* strains were inoculated and grown as above. The culture broths were filtered through Miracloth (Merck Millipore, Darmstadt, Germany). The mycelia were washed with water twice, dehydrated with tert-butanol, lyophilized, and coated with platinum-vanadium. Mycelia were observed under a Hitachi SU8000 scanning electron microscope (Hitachi, Tokyo, Japan) at an accelerating voltage of 3 kV.

### Visualization of Biofilms

Biofilms were visualized by using the method of Gravelat et al. (2010), with some modifications. Conidia (final concentration, 1 × 10^5^/ml) of the wild-type, AGΔ, GAGΔ, and AG-GAGΔ *A. oryzae* strains were inoculated in 10 ml of CDE medium on a polystyrene plate (internal diameter 60 mm) and incubated for 24 h at 30°C. Spent culture medium was removed from the plate, and the plate was then washed three times with PBS. Then 5 ml of 0.5% (w/v) crystal violet solution was added to the plate, which was incubated at room temperature for 5 min. Excess stain was removed, and the plate was washed twice with water. The visualized biofilm was imaged by using a flatbed scanner (GT-X820; Seiko Epson Corp., Nagano, Japan).

### Assay for Cell Wall Susceptibility to Lysing Enzymes

Susceptibility of the fungal cell wall to Lysing Enzymes (LE), a commercial preparation containing β-1,3-glucanase and chitinase (Sigma, St. Louis, MO, USA), was assayed as described previously (Yoshimi et al., 2013). Washed 1-day-old mycelia of the wild-type, AGΔ, GAGΔ, and AG-GAGΔ strains (30 mg fresh weight) grown in CDE medium at 30°C were suspended in 1 ml of 0.8 M NaCl in sodium phosphate buffer (10 mM, pH 6.0) containing 10 mg/ml LE and incubated for 1, 2, or 4 h at 30°C. The number of protoplasts generated from the mycelia was counted with a hemocytometer (A106, SLGC, Tokyo, Japan).

### Assay for Growth Inhibition by Congo Red

Sensitivity of the wild-type, AGΔ, GAGΔ, and AG-GAGΔ strains to Congo Red was evaluated by using our previously described method (Yoshimi et al., 2013), with a minor modification. Briefly, conidial suspensions of each strain (1.0 × 10^4^ cells) were spotted on the centers of CDE plates containing Congo Red (10, 20, 40, 80, or 120 μg/ml) and incubated at 30°C for 3 days. The dose response was determined by plotting the mean diameters of the colonies on media with Congo Red as a percentage of those on control medium. Each experiment was performed in quadruplicate.

### Fractionation of Cell Wall Components and Quantification of Carbohydrate Composition

Conidia (final concentration, 1.0 × 10^5^/ml) of the wild-type, AGΔ, GAGΔ, and AG-GAGΔ strains were inoculated into 200 ml of YPD medium in 500-ml Erlenmeyer flasks and rotated at 120 rpm at 30°C. Mycelia were collected by filtration through Miracloth, washed twice with 20 ml of water, and lyophilized. Mycelia were pulverized with a MM400 bench-top mixer mill (Retsch, Haan, Germany), and the resulting powder (1 g) was suspended in 40 ml of 0.1 M sodium phosphate buffer (pH 7.0). Cell wall components were fractionated by hot-water and alkali treatment (Miyazawa et al., 2016); the fractionation resulted in hot-water-soluble (HW), alkali-soluble (AS), and alkali-insoluble (AI) fractions. The AS fraction was further separated into a fraction soluble in water at neutral pH (AS1) and an insoluble fraction (AS2). The carbohydrate composition of the fractions was quantified as described previously (Yoshimi et al., 2013). Briefly, 10 mg of each cell wall fraction was hydrolyzed with sulfuric acid and then neutralized with barium sulfate. The carbohydrate composition of the hydrolysate was determined by using high-performance anion-exchange chromatography (HPAEC). For GalN quantification, the carbohydrate composition of sulfuric acid-hydrolyzed HW fractions (50 mg each) was quantified.

### Purification of Galactosaminogalactan From Culture Supernatant of the α-1,3-Glucan-Deficient Strain by Fractional Precipitation With Ethanol

Conidia (final concentration, 1.0 × 10^6^/ml) of the AGΔ or AG-GAGΔ strain (negative control) were inoculated into three flasks, each containing 1 L of modified Brian medium (Fontaine et al., 2011), and rotated at 160 rpm at 30°C for 72 h. The mycelia were removed by filtration through Miracloth. The supernatants were combined and concentrated to 1 L by evaporation, dialyzed against water at 4°C, and concentrated again to 1 L; then, 20 g of NaOH was added (final concentration, 0.5 M) at 4°C with stirring. The mixture was centrifuged at 3000 ×*g* at 4°C for 10 min and a pellet was obtained (referred to hereafter as the 0 vol). EtOH (0.5 L) was added to the supernatant and the mixture was incubated for 5 h at 4°C with stirring, then centrifuged at 3000 ×*g* at 4°C for 10 min, and a pellet (0.5-vol. EtOH fraction) was obtained. These procedures were repeated to obtain 1-, 1.5-, 2-, and 2.5-vol. EtOH fractions. Each fraction was neutralized with 3 M HCl, dialyzed against water, and freeze-dried. The carbohydrate composition of each fraction was determined as above. For mycelial aggregation assay, each freeze-dried EtOH fraction from the AGΔ strain (2 mg) and the 1.5-vol. EtOH fraction from the AG-GAGΔ strain were dissolved in 1 ml of 0.1 M HCl and vortexed for 10 min.

### Conidial and Mycelial Aggregation Assay

A modified method of Fontaine et al. (2010) was used. Conidia (5 × 10^5^) were inoculated into 500 μl of CDE liquid medium containing 0.05% Tween 20 in a 48-well plate and agitated at 1,200 rpm with a microplate mixer (NS-P; As One, Osaka, Japan) at 30°C for 3, 6, or 9 h. Conidial aggregates were then examined under a stereomicroscope (M125; Leica Microsystems, Wetzlar, Germany). Mycelial aggregation in the presence of GAG was evaluated as follows. Conidia (final concentration, 1.0 × 10^7^/ml) of the AG-GAGΔ strain were inoculated into 50 ml of YPD medium and rotated at 120 rpm at 30°C for 9 h. The mycelia were collected by filtration through Miracloth and washed twice with water. The mycelia (wet weight, 500 mg) were resuspended in 10 ml of PBS, and the suspension (25 μl) was added into a mixture of 400 μl of water, 50 μl of 1 M sodium phosphate buffer (pH 7.0), and 25 μl of the EtOH fraction (from AGΔ) or the mock fraction (from AG-GAGΔ). Aggregates were examined under a stereomicroscope after 1 h.

An aggregation assay in the presence of the AS2 fraction was performed by using the following method. The AS2 fraction (10 mg) was dissolved in 1 M NaOH (100 μl). Then, an aliquot of water (900 μl) was mixed into the solution (AS2 fraction concentration, 10 mg/ml). Mycelial suspension (25 μl) was added to a mixture of 400 μl of water, 50 μl of 1 M sodium phosphate buffer (pH 7.0), and 25 μl of the solution containing the AS2 fraction. Aggregates were examined under a stereomicroscope after 1 h.

To quantify the conidial aggregation, the total number of conidia was counted by observing microscopic images (× 1,000). Then the aggregated conidia were counted in the same images. Aggregated conidia were defined as aggregates in which more than five conidia were gathered. The aggregation percentage was determined by observing at least 100 conidia.

To evaluate the effect of pH on mycelial aggregation by GAG, mycelial suspension (25 μl) was added to a mixture of 450 μl of buffer (final concentration, 100 mM) and 25 μl of the 1.5-vol. EtOH fraction. The following buffers were used: pH 4.0–5.0, sodium acetate; pH 6.0–7.0, sodium phosphate; pH 8.0, Tricine-NaOH. Aggregates were examined after 1 h.

To examine the effect of inhibiting hydrogen bond formation, mycelial suspension (25 μl) was added to a mixture of 450 μl of 100 mM sodium phosphate buffer (pH 7.0) and 0, 1, 2, 4, or 8 M urea, and 25 μl of the 1.5-vol. EtOH fraction.

### Visualization of α-1,3-Glucan and Galactosaminogalactan in the Cell Wall

Germinating conidia cultured in a 48-well plate were dropped onto a glass slide, washed twice with PBS, and fixed with 4% (w/v) paraformaldehyde for 10 min. Samples were washed twice with 50 mM potassium phosphate buffer (pH 6.5) and stained at room temperature for 2 h with Alexa Fluor 647-conjugated soybean agglutinin (SBA; 100 μg/ml) (Invitrogen) and α-1,3-glucanase-α-1,3-glucan-binding domain fused with GFP (AGBD-GFP; 100 μg/ml) (Suyotha et al., 2013) in 50 mM phosphate buffer (pH 6.5). After being washed with the same buffer, the samples were imaged under a FluoView FV1000 confocal laser-scanning microscope (Olympus, Tokyo, Japan). Cells were then washed three times with PBS, and a drop of PBS containing secondary antibody (anti-rabbit IgG antibody-Alexa Fluor 568 conjugate; Invitrogen) was added. The sample was incubated at room temperature for 1 h, washed as above, and imaged under a confocal laser-scanning microscope.

### Acetylation of the Amino Group of Galactosaminogalactan

The 1.5-vol. EtOH fraction from the AGΔ (5 mg) was dissolved in ice cold 0.5 M NaOH (800 μl), neutralized with ice cold 2 M HCl, and then added to 4 ml of 50 mM sodium acetate. Then, methanol (4 ml) and acetic anhydrate (10 mg) were added and the mixture was stirred at room temperature for 24 h. The sample was then evaporated, washed three times with methanol, dialyzed against water, and freeze-dried. The procedure was then repeated.

### Determination of Degree of Deacetylation of Galactosaminogalactan by Colloidal Titration

The degree of deacetylation (DD) of GAG was determined by colloidal titration in accordance with the method used to determine the DD of chitosan (Terayama, 1951; Senju, 1969; Hattori et al., 2009). Freeze-dried GAG (5 mg) was dissolved in 0.1 M HCl, and the solution was then made up to 1 g with 0.1 M HCl. Water (30 ml) was added to the GAG solution, and the mixture was stirred thoroughly. A few drops of 0.1% (w/v) toluidine blue solution as an indicator were added to the mixture. The GAG solution was titrated with N/400 potassium polyvinylsulfate (PVSK). The endpoint of the titration was determined by the color change of the indicator from blue to red. The DD of GalNAc in the 1.5-vol. EtOH fraction from AGΔ was determined by using the following equations:

$$DD\left(\%\right)=\frac{\frac{X}{161}}{\frac{X}{161}+\frac{Y}{203}}\times 100$$

$$X=\frac{1}{400}\times \frac{1}{1000}\times f\times 161\times v$$

Y=a×b−X

where:

*a*, sample (g); *b*, ratio of total hexosamine in 1.5-vol. EtOH fraction from AGΔ; *v*, titer of N/400 PVSK solution (ml); *f*, factor of N/400 PVSK solution.

The DD of GalNAc residues of the sample was determined by these equations. The ratio of total hexosamine in the 1.5-vol. EtOH fraction was determined with *p*-(dimethylamino)-benzaldehyde reagent after 4 h of 8 N HCl hydrolysis at 100°C using galactosamine as a standard (Johnson, 1971; Fontaine et al., 2011). For considering non-GAG components in the sample, the titer background (1.5-vol. EtOH fraction from AG-GAGΔ titrated with N/400 PVSK) was subtracted from the titer of each sample.

### Quantification of CutL1 Production

A *cutL1*-overexpressing strain (AG-GAGΔ-cutL1) was constructed as described previously (Miyazawa et al., 2016) with the pNGA-gla-Cut plasmid (Maeda et al., 2005). Integration of a single copy of the *cutL1-*overexpression construct at the *niaD* locus was confirmed by Southern blot analysis (Supplementary Figure S2). Enzyme production in the mutants was evaluated as described previously (Miyazawa et al., 2016), with some modifications. Briefly, conidia (final concentration, 1 × 10^4^/ml) of the WT-cutL1, AGΔ-cutL1, and AG-GAGΔ-cutL1 strains were inoculated into 50 ml of YPM medium and rotated at 100 rpm at 30°C for 24 h. The culture broth was filtered through Miracloth. Mycelial cells were dried at 70°C for 24 h and weighed. Proteins were precipitated from an aliquot of the filtrate (400 μl) with 100% (w/v) trichloroacetic acid (200 μl), separated by SDS-PAGE, and stained with Coomassie Brilliant Blue. ImageJ software was used to quantify the CutL1 in the broth; purified CutL1 was used for calibration.

### ^13^C NMR Analysis of Cell Wall Fractions

^13^C NMR analysis was performed as described previously (Miyazawa et al., 2018). The AS2 fractions from the wild-type and GAGΔ strains were dissolved in 1 M NaOH/D_2_O. Me_2_SO-d_6_ (deuterated dimethyl sulfoxide; 5 μl) was added to each sample. ^13^C NMR spectra were obtained by using a JNM-ECX400P spectrometer (JEOL, Tokyo, Japan) at 400 MHz, 35°C (72,000 scans).

### RNA Purification, Reverse Transcription, and Quantitative Polymerase Chain Reaction

Total RNA was extracted by using Sepasol-RNA I Super according to the manufacturer’s instructions (Nacalai Tesque, Kyoto, Japan). Total RNA (2 μg) was reverse transcribed and cDNA was amplified by using a High-Capacity cDNA Reverse Transcription Kit according to the manufacturer’s instructions (Thermo Fisher Scientific, Waltham, MA, USA). Quantitative PCR was performed with the primers AoagsA-RT-F and AoagsA-RT-R, AoagsB-RT-F and AoagsB-RT-R, and AoagsC and AoagsC-RT-R (Table 2), in sequence, using KOD SYBR qPCR Mix (Toyobo Co., Ltd., Osaka, Japan).

### Statistical Analysis

Student’s *t*-test was used for the comparison of paired samples, and Tukey’s test was used to compare multiple samples.

## Results

### *Aspergillus oryzae* Has a Galactosaminogalactan Biosynthetic Gene Cluster

**Figure 1:**
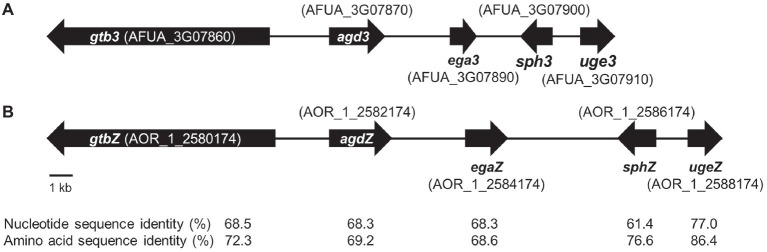
GAG biosynthetic cluster in **(A)** *Aspergillus fumigatus* and **(B)** *Aspergillus oryzae.* The cluster of *A*. *oryzae* was predicted from the sequence of the cluster of *A. fumigatus* by using a BLAST search.

In *A. fumigatus*, GAG biosynthesis is regulated by a cluster of five genes, and this cluster is conserved in a wide range of filamentous fungi (Lee et al., 2016). To check whether *A. oryzae* possesses the GAG gene cluster, we used a BLAST search^1^. We found that all five GAG biosynthetic genes, orthologous to *A. fumigatus uge3*, *sph3*, *ega3*, *agd3*, and *gtb3* (Figure 1A), are conserved in *A. oryzae*: *ugeZ*, *sphZ*, *egaZ*, *agdZ*, and *gtbZ* (Figure 1B). *Aspergillus oryzae* UgeZ had motifs conserved among group 2 epimerases (Lee et al., 2014), SphZ contained a spherulin 4 conserved region (Bamford et al., 2015), and AgdZ had the conserved motifs of the carbohydrate esterase family 4 (Lee et al., 2016). These findings indicated that *A*. *oryzae*, similar to *A. fumigatus*, can produce GAG.

### Hyphae of the AG-GAGΔ Strain Are Completely Dispersed in Liquid Culture

**Figure 2:**
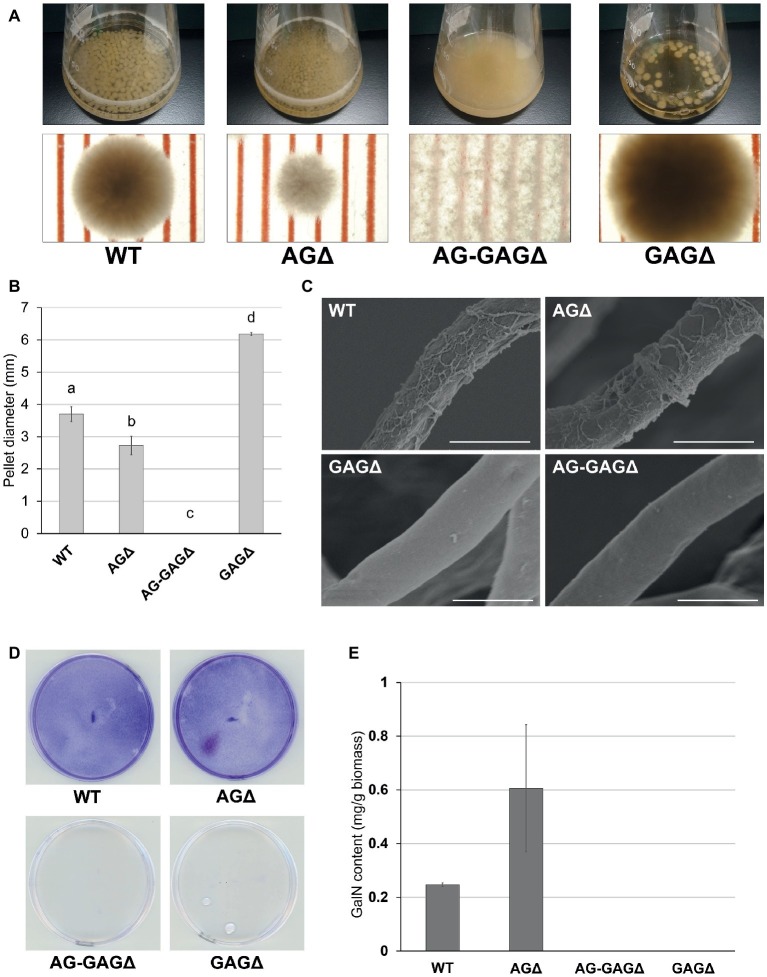
Phenotypes of *Aspergillus oryzae* Δ*agsA*Δ*agsB*Δ*agsC*Δ*sphZ*Δ*ugeZ* (AG-GAGΔ) and Δ*sphZ*Δ*ugeZ* (GAGΔ) strains in liquid culture. **(A)** The wild-type (WT), Δ*agsA*Δ*agsB*Δ*agsC* (AGΔ), AG-GAGΔ, and GAGΔ strains were cultured in Erlenmeyer flasks (upper row), and images of hyphal pellets were taken under a stereomicroscope (bottom row; scale, 1 mm) at 24 h of culture. **(B)** The mean diameter of hyphal pellets was determined by measuring 10 randomly selected pellets per replicate under a stereomicroscope. Error bars represent standard deviations calculated from three replicates. Different letters indicate significant differences within each condition by Tukey’s test (*p* < 0.05). **(C)** Morphology of each strain was examined under a scanning electron microscope. Scale bars, 5 μm. **(D)** Biofilm formation on polystyrene plates. Each strain was cultured for 24 h on a polystyrene plate (internal diameter 6 cm), after which the biofilms were washed and stained with crystal violet. **(E)** Galactosamine (GalN) content in the hot water-soluble fraction of the cell wall from the WT, AGΔ, AG-GAGΔ, and GAGΔ strains. Error bars represent standard error of the mean calculated from three replicates.

Because disruption of the *sph3* and *uge3* genes leads to a loss of GAG in *A. fumigatus* (Lee et al., 2014; Bamford et al., 2015), we disrupted *sphZ* and *ugeZ* in *A. oryzae* in the genetic background of the wild-type and AGΔ strains, and we obtained the GAGΔ and AG-GAGΔ strains, respectively. The wild-type, AGΔ, AG-GAGΔ, and GAGΔ strains showed almost the same mycelial growth and conidiation on CD agar plates after 5 days at 30°C (Supplementary Figure S3). When grown in YPD liquid medium at 30°C for 24 h, the wild-type strain formed significantly larger hyphal pellets (3.7 ± 0.2 mm in diameter) than did the AGΔ strain (2.7 ± 0.3 mm; Figures 2A,B), in good agreement with our previous results (Miyazawa et al., 2016). The hyphae of the AG-GAGΔ strain were completely dispersed, and the GAGΔ strain formed significantly larger hyphal pellets (6.2 ± 0.0 mm) than did the wild-type strain (Figures 2A,B). These results strongly suggest that, in addition to α-1,3-glucan, GAG has a role in hyphal adhesion in *A. oryzae* and that the defect in both α-1,3-glucan and GAG biosynthetic genes is required for full dispersion of *A. oryzae* hyphae.

Scanning electron microscopy revealed that the surface of *A. fumigatus* hyphae has GAG-dependent decorations in liquid culture; these are lost in the *sph3*, *uge3*, and *agd3* gene disruptants (Lee et al., 2014, 2016; Bamford et al., 2015). We investigated whether the hyphae of *A. oryzae* GAGΔ and AG-GAGΔ strains lack such decorations. As expected, we observed fibrous decorations on the hyphal cells of the wild-type and AGΔ *A. oryzae* strains (Figure 2C), but the hyphae of the GAGΔ and AG-GAGΔ strains had smooth surfaces (Figure 2C). These results suggest that the fibrous decorations on the cell surface are attributable to the presence of the GAG biosynthetic gene cluster in *A. oryzae*. A defect in GAG led to a loss of formation of adherent biofilm on solid surfaces (Figure 2D).

Gravelat et al. (2013) quantified the GAG content as the amount of GalN after complete hydrolysis of ethanol-precipitated supernatant from *A. fumigatus* culture. To apply this approach to *A. oryzae*, we analyzed the hydrolyzed HW fractions of each strain by HPAEC. The HW fractions from both the wild-type and AGΔ strains contained 0.2–0.3 mg/g biomass GalN (Figure 2E), whereas GalN was hardly detectable in the HW fractions from the GAGΔ and AG-GAGΔ strains (Figure 2E). These results show that *ugeZ* or *sphZ*, or both, are essential for GAG production in *A. oryzae*.

We used three approaches to analyze why the GAGΔ strain formed larger hyphal pellets in liquid culture: (1) HPAEC-pulsed amperometric detection analysis of cell wall components in alkali-soluble fractions showed no significant difference in the amount of glucose in the AS2 fractions between the wild-type and GAGΔ strains (Supplementary Figure S4A). The other cell wall components were similar among the four strains, except for the amount of glucose in the AS2 fractions (Supplementary Figure S4A). (2) Expression of the *agsB* gene, which encodes the main α-1,3-glucan synthase of *A*. *oryzae* (Zhang et al., 2017), was slightly lower in the GAGΔ strain than in the wild-type strain at 6 h of culture, but it was slightly higher at 24 h (Supplementary Figure S4B). The *agsA* and *agsC* genes, which encode minor α-1,3-glucan synthases, were scarcely expressed at 6 h (Supplementary Figure S4B). (3) ^13^C NMR analysis of the AS2 fraction showed that the main component was α-1,3-glucan in both strains (Supplementary Figure S4C). (4) The AS2 fraction from the wild-type or GAGΔ strain was added to the hyphae of the AG-GAGΔ strain, resulting in the formation of similar hyphal aggregates in both samples; no aggregate formation was observed in the presence of the AS2 fraction from the AGΔ or the AG-GAGΔ strain (Supplementary Figure S4D). The reason why the GAGΔ strain formed larger aggregated pellets remains unclear from the results of our experiments.

### Disruptants of AG and Galactosaminogalactan Biosynthetic Genes Are Sensitive to Lysing Enzymes and Congo Red

**Figure 3:**
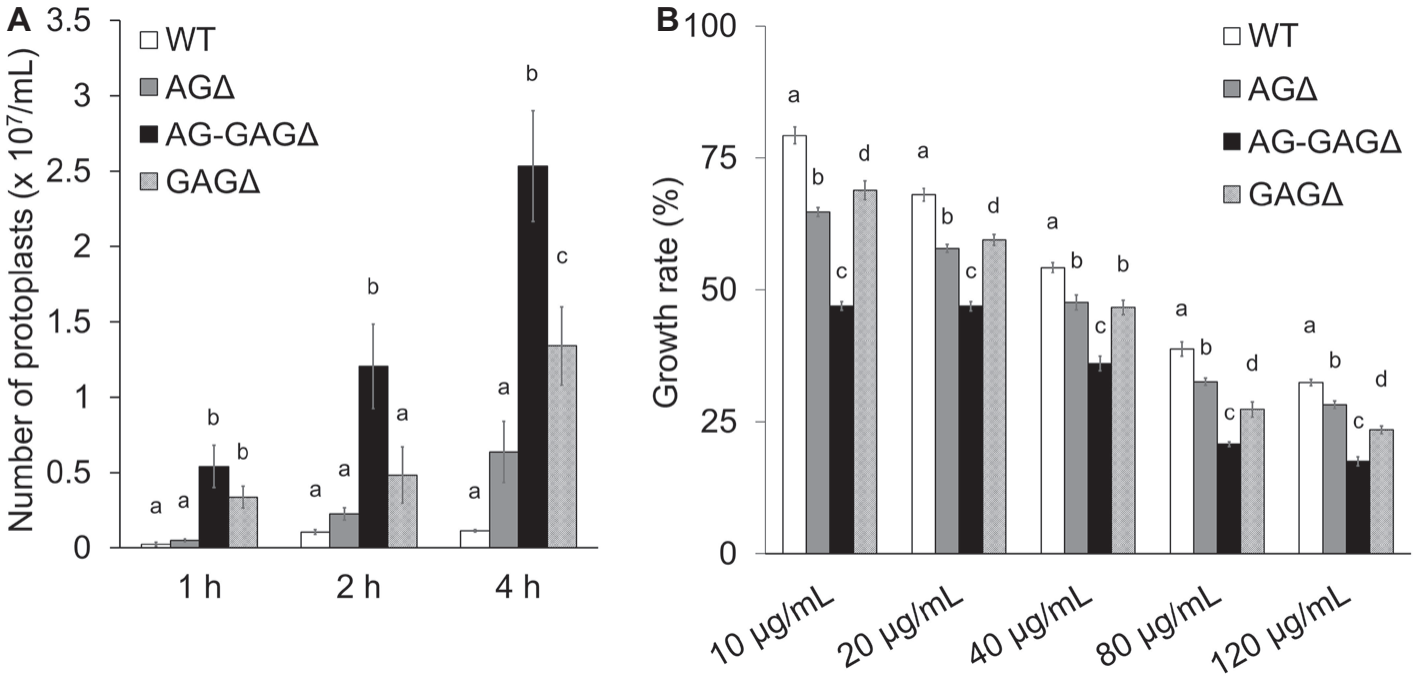
Sensitivity to Congo Red and Lysing Enzymes. **(A)** Mycelia cultured for 1 day were suspended in sodium phosphate buffer (10 mM, pH 6.0) containing 0.8 M NaCl and 10 mg/ml Lysing Enzymes. After 1, 2, and 4 h, protoplasts were counted under a microscope. Error bars represent the standard deviation calculated from three replicates. **(B)** Growth rates after 3 days on CDE medium at the indicated concentrations of Congo Red. Diameter of the colonies grown on CDE medium without Congo Red was considered as 100%. Error bars represent standard deviations calculated from three replicates. In both panels, different letters indicate significant differences within each condition by Tukey’s test (*p* < 0.05).

To investigate the consequences of cell wall alteration caused by the loss of GAG, we assessed the susceptibility of the wild-type, AG∆, AG-GAG∆, and GAG∆ strains to LE and CR. The concentrations of protoplasts formed from hyphae tended to be higher for the AG∆ strain than for the wild-type strain after 2 and 4 h of treatment with LE (0.05 < *p* < 0.1; Figure 3A). In contrast, the protoplast concentration was significantly higher for the AG-GAG∆ strain than for the wild-type and AG∆ strains at each time point (Figure 3A). The protoplast concentration was also significantly higher for the GAG∆ strain than for the wild-type and AG∆ strains after 1 and 4 h (Figure 3A), but it was significantly lower than for the AG-GAG∆ strain after 4 h (Figure 3A). All three mutant strains were significantly more sensitive to CR than the wild type: the AG-GAG∆ strain was most sensitive, and the AG∆ and GAG∆ strains showed similar sensitivity (Figure 3B). These data revealed that α-1,3-glucan and GAG additively contribute to cell wall protection from cell wall-degrading enzymes and environmental chemicals.

### Temporally and Spatially Different Contributions of α-1,3-Glucan and Galactosaminogalactan to Hyphal Aggregation in Liquid Culture

**Figure 4:**
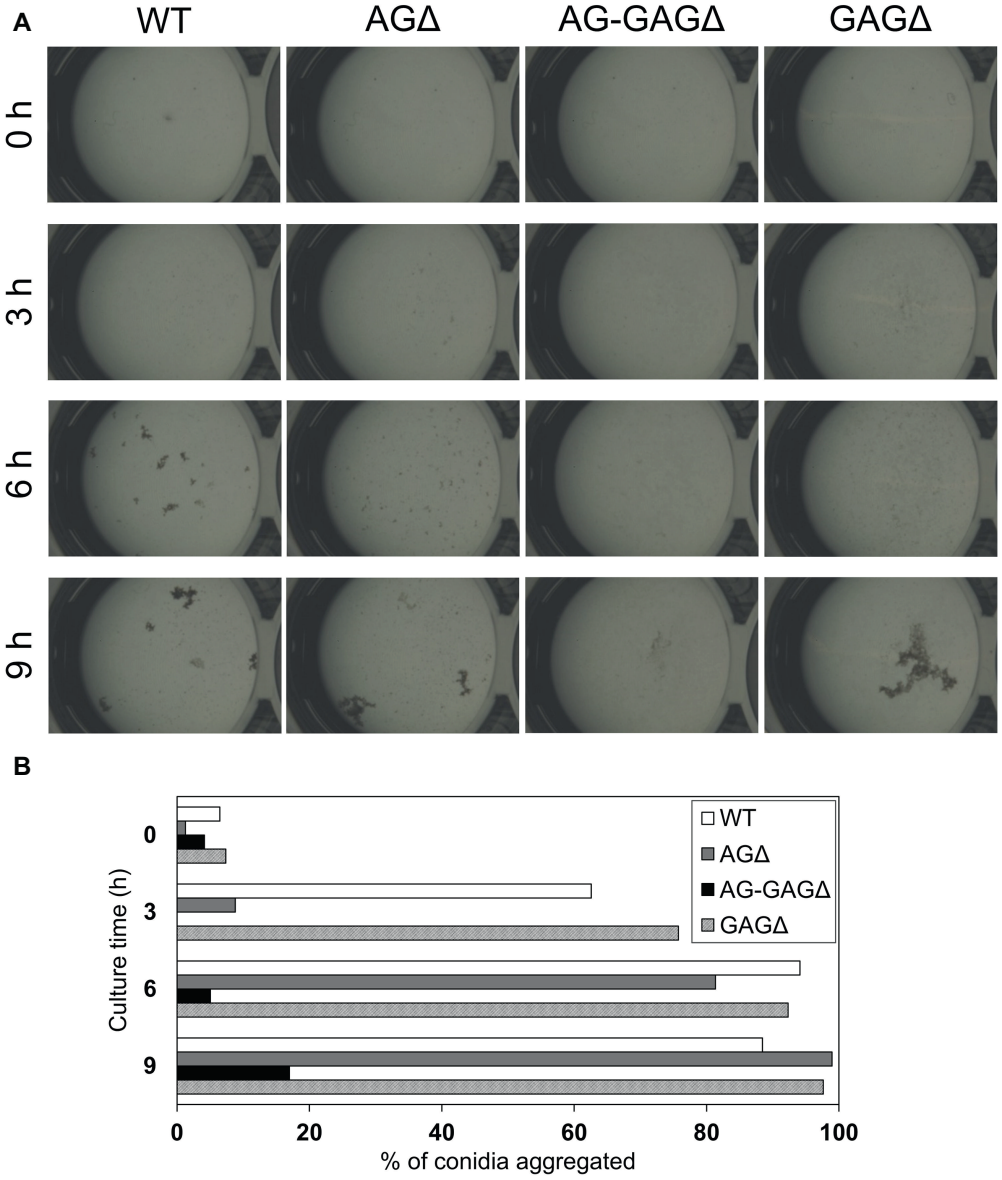
Conidial aggregation assay. **(A)** Conidia (5 × 10^5^) of the wild-type (WT), Δ*agsA*Δ*agsB*Δ*agsC* (AGΔ), Δ*agsA*Δ*agsB*Δ*agsC*Δ*sphZ*Δ*ugeZ* (AG-GAGΔ), and Δ*sphZ*Δ*ugeZ* (GAGΔ) strains were inoculated into 500 μl of CDE liquid medium and incubated at 30°C with shaking (1,200 rpm). Photographs were taken at the indicated time points under a stereomicroscope (magnification, ×8). **(B)** Percentage conidial aggregation at indicated culture times.

**Figure 5:**
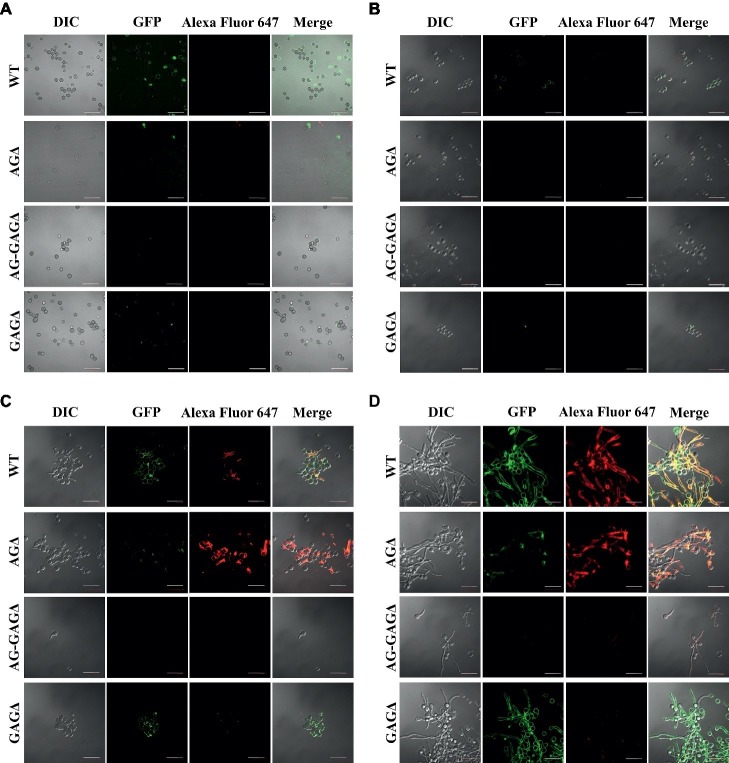
Visualization of AG and GAG in the cell wall by staining with AGBD-GFP and lectin (soybean agglutinin). Conidia (5.0 × 10^5^) of the wild-type (WT), Δ*agsA*Δ*agsB*Δ*agsC* (AGΔ), Δ*agsA*Δ*agsB*Δ*agsC*Δ*sphZ*Δ*ugeZ* (AG-GAGΔ), and Δ*sphZ*Δ*ugeZ* (GAGΔ) strains were inoculated into 500 μl of CDE liquid medium and incubated at 30°C for **(A)** 0, **(B)** 3, **(C)** 6, and **(D)** 9 h with shaking (1,200 rpm). At each time point, the cells were dropped on a glass slide, fixed with 4% (w/v) paraformaldehyde, stained with AGBD-GFP and soybean agglutinin-Alexa Fluor 647 conjugate (100 μg/ml each), and observed under a confocal laser-scanning microscope (×1,000). Scale bars, 20 μm.

The complete dispersion of the AG-GAGΔ hyphae demonstrated that both α-1,3-glucan and GAG function as adhesive factors for hyphal aggregation in *A. oryzae*, and consequently the hyphae expressing both polysaccharides form pellets (Figure 2). To analyze the temporal and spatial contribution of the two polysaccharides, wild-type, AGΔ, GAGΔ, and AG-GAGΔ conidia were cultured in 48-well plates, and formation of hyphal pellets was examined (Figure 4A). The presence of α-1,3-glucan and GAG on the surfaces of conidia and germinating hyphae in liquid culture was analyzed by fluorescence microscopy with AGBD-GFP, which binds specifically to α-1,3-glucan, and lectin SBA, which binds specifically to GalNAc (Figure 5). At the initiation of culture (0 h), conidia of all strains formed scarce aggregates (Figures 4A,B). Fluorescence of AGBD-GFP was observed on wild-type and GAGΔ conidia, but not on AGΔ or AG-GAGΔ conidia (Figure 5A). SBA fluorescence was undetectable on conidia of any strains (Figure 5A). At 3 h after inoculation, 63 and 76%, respectively, of swollen conidia of the wild-type and GAGΔ strains had aggregated and formed small pellets, but aggregates of AGΔ and AG-GAGΔ swollen conidia were scarce (Figures 4A,B). At 3 h, AGBD-GFP fluorescence was detectable on wild-type and GAGΔ germinated conidia, but none of the strains was stained with SBA (Figure 5B). At 6 h, the wild-type, AGΔ, and GAGΔ formed hyphal pellets, but aggregates of AG-GAGΔ were scarce (Figure 4A). Aggregation reached nearly 95% in the wild-type and GAGΔ, 80% in AGΔ, and 0% in AG-GAGΔ (Figure 4B). AGBD-GFP fluorescence was observed on hyphae of the wild-type and GAGΔ, and that of SBA was observed in the wild-type and AGΔ strains (Figure 5C). At 9 h, the wild-type, GAGΔ, and AGΔ strains formed hyphal pellets (Figure 4A) similar to those formed after 24 h of culture in YPD medium. Aggregation of germinating conidia reached about 90%, except in AG-GAGΔ (Figure 4B). The fluorescence profiles of AGBD-GFP and SBA for each strain were similar to those observed at 6 h (Figure 5D). The AG-GAGΔ strain hardly formed any hyphal pellets at any time point (Figures 4, 5). Neither conidia nor hyphae of AG-GAGΔ were stained by AGBD-GFP or SBA (Figure 5). These results indicate that hyphal aggregation caused by α-1,3-glucan was initiated just after inoculation, whereas GAG-dependent hyphal aggregation started 3–6 h after inoculation.

### Galactosaminogalactan-Dependent Aggregation of Hyphae *in vitro* and Its pH Dependence

**Figure 6:**
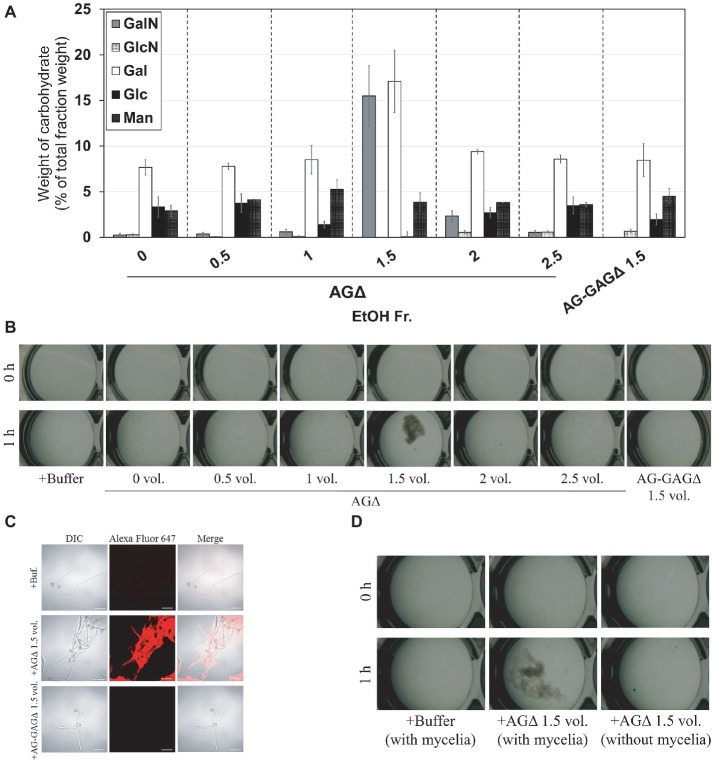
Aggregation of mycelia of the AG-GAGΔ strain induced by ethanol-precipitated GAG. **(A)** Monosaccharide composition of the fractions obtained from culture supernatant by ethanol precipitation. **(B)** Mycelial suspension of the AG-GAGΔ strain (25 μl) was added into a mixture of 400 μl of water, 50 μl of 1 M sodium phosphate buffer (pH 7.0), and 25 μl of 2 mg/ml of the fractions prepared from the AGΔ or AG-GAGΔ strains, as indicated. Samples were incubated at 30°C for 1 h with shaking and examined under a stereomicroscope (magnification, ×8). **(C)** Mycelia incubated for 1 h in the presence of EtOH-precipitated GAG were stained with soybean agglutinin-Alexa Flour 647 conjugates and observed under a confocal laser-scanning microscope (×1,000). Scale bars, 20 μm. **(D)** Aggregation assay with the 1.5-vol. fraction from AGΔ was performed as in **(A)**, with or without mycelial suspension of AG-GAGΔ.

According to the previously described GAG purification method (Fontaine et al., 2011), we obtained the ethanol precipitates from the AGΔ strain and washed them with 150 mM sodium chloride. However, the precipitates were fully solubilized in 150 mM sodium chloride. Therefore, we developed an EtOH fractional precipitation method to isolate GAG from culture supernatants, and we obtained six fractions. The 0-, 0.5-, 1-, 2-, and 2.5-vol. fractions from the AGΔ strain contained approximately 8% of Gal and 5% of mannose, with a small amount of GalN (Figure 6A). The 1.5-vol. fraction from the AGΔ strain contained 16% of GalN, 17% of Gal, and 4% of mannose (Figure 6A); thus, this fraction but not the other fractions appeared to contain mainly GAG and galactomannan. The 1.5-vol. fraction from the AG-GAGΔ strain contained no GalN but contained 8% of Gal and 5% of mannose (Figure 6A). As calculated from the composition of the 1.5-vol. fraction from the AG-GAGΔ strain, the 1.5-vol. fraction from AGΔ appeared to contain approximately 25% of GAG. To evaluate whether the aggregation of hyphae could be reproduced *in vitro*, the fractions were added to the mycelia of the AG-GAGΔ strain and mycelial aggregation was examined. Only the 1.5-vol. fraction from the AGΔ strain induced aggregation (Figure 6B). The aggregates were stained with an SBA-Alexa Fluor 647 conjugate (Figure 6C). The 1.5-vol. fraction from AGΔ did not form aggregates without mycelia (Figure 6D). The DD of GalNAc residues of GAG in the 1.5-vol. fraction, as determined by colloidal titration, was 48.9 ± 4.6% (Table 3).

**Table 3:** Degrees of deacetylation of *N*-acetylgalactosamine residues of galactosaminogalactan.

Sample	Degree of deacetylation (%)
AGΔ 1.5-vol. EtOH Fr.	48.9 ± 4.6
AGΔ 1.5-vol. EtOH Fr. (acetylated)	2.0 ± 0.5*
AGΔ 1.5-vol. EtOH Fr. (non-acetylated)	43.6 ± 5.2

*Values represent the mean ± standard error of the three replicates. Asterisk indicates significant difference (p < 0.01) with the AGΔ 1.5-vol. EtOH Fr. (non-acetylated)*.

**Figure 7:**
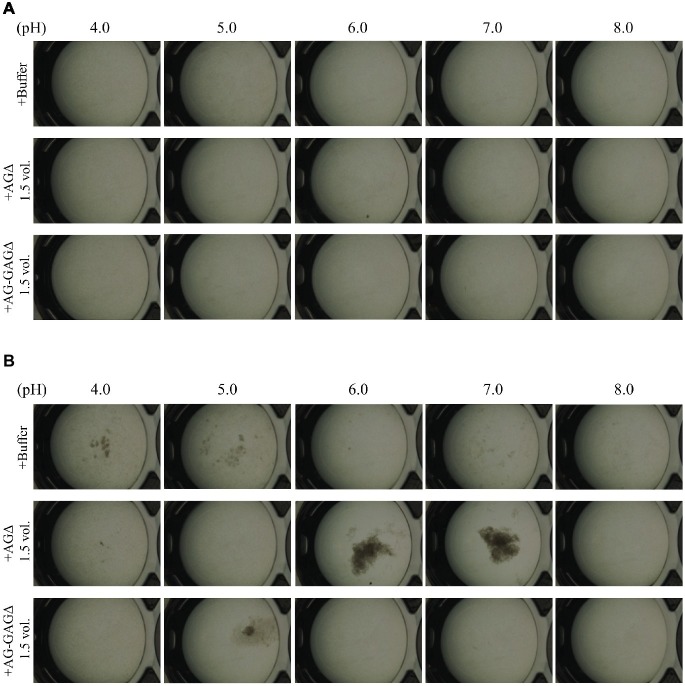
pH-dependence of GAG aggregation. Mycelial suspension of the AG-GAGΔ strain (25 μl) was added to 450 μl of buffers with different pH and 25 μl of the 1.5-vol. EtOH fraction prepared from the AGΔ or AG-GAGΔ strain as indicated. Samples were incubated at 30°C for **(A)** 0 h and **(B)** 1 h with shaking and examined under a stereomicroscope (magnification, ×8).

In *A. fumigatus*, GalNAc moieties in GAG are partly deacetylated and consequently positively charged (Fontaine et al., 2011), and we wondered whether GAG-dependent aggregation depends on pH. Addition of GAG to mycelia of the AG-GAGΔ strain led to aggregation at pH 6, 7; aggregates were scarce at pH 4, 5, and 8 (Figure 7A). The conidia remained dispersed upon the addition of the mock fraction at pH 4, 6, 7, and 8 (Figure 7B). At pH 5, slight aggregates were formed when the mock fraction was added (Figure 7B). In addition, although the degree of aggregation was very low compared with that in the presence of GAG at neutral pH, the AG-GAGΔ hyphae formed slight, or very small, aggregates at pH 4 and 5 in the absence of GAG or the mock fraction (Figure 7B). There might therefore still be some unknown aggregation factor working at low pH in AG-GAGΔ hyphae. These results suggest that the increased positive charge in GAG at acidic pH leads to electric repulsion among GAG chains and consequently prevents GAG-dependent mycelial aggregation. Around the neutral pH, the positive charge might be lower and consequently GAG might contribute to hyphal adhesion *via* non-electrostatic interactions. The reason for the absence of aggregate formation at pH 8 is unknown.

### Galactosaminogalactan-Dependent Aggregation Is Caused by Hydrogen Bonding Between Polysaccharides

**Figure 8:**
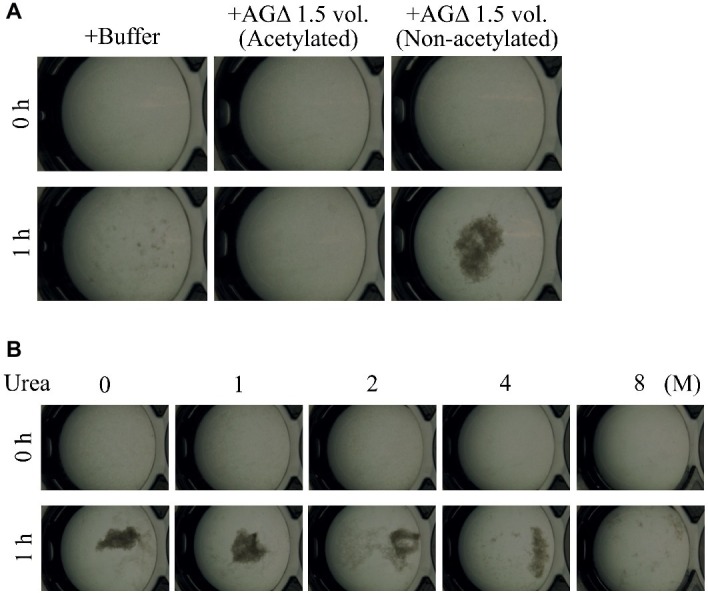
Mycelial aggregation in the presence of **(A)** acetylated GAG or **(B)** urea. **(A)** The amino groups of ethanol-precipitated GAG were acetylated with acetic anhydrate. Mycelial suspension of the AG-GAGΔ strain (25 μl) was added to a mixture of 450 μl of 100 mM sodium phosphate buffer (pH 7.0) and 25 μl of the 1.5-vol. EtOH fraction prepared from AGΔ (acetylated or not). **(B)** Mycelial suspension of the AG-GAGΔ strain (25 μl) was added to a mixture of 450 μl of 100 mM sodium phosphate buffer (pH 7.0) containing 0, 1, 2, 4, or 8 M urea, and 25 μl of the 1.5-vol. EtOH fraction prepared from the AGΔ strain. Samples were incubated at 30°C for 1 h with shaking and examined under a stereomicroscope (magnification, ×8).

We hypothesized that GAG-dependent aggregation was caused by hydrogen bonding *via* the amino groups of GalN. To test this hypothesis, we treated the 1.5-vol. EtOH fraction with (acetylation) or without acetic anhydrate and then evaluated the aggregation. In the presence of non-*N*-acetylated GAG, the AG-GAGΔ mycelia aggregated, similar to the results in Figure 6A, whereas GAG *N*-acetylation weakened mycelial aggregation (Figure 8A). The DD of the *N*-acetylated 1.5-vol. fraction was 2.0 ± 0.5%, which was significantly smaller than that of the non-*N*-acetylated 1.5-vol. fraction (43.6 ± 5.2%; *p* < 0.01) (Table 3). These results suggest that the amino groups of GalN are involved in GAG-dependent aggregation.

To confirm that GAG-dependent aggregation relies on hydrogen bonds, we performed mycelial aggregation assay in the presence of urea, which breaks hydrogen bonds. Mycelia aggregated without urea, but aggregation was weakened by increasing urea concentrations (Figure 8B). Taken together, these results strongly suggest that hydrogen bond formation *via* the amino groups of GalN is important for GAG-dependent aggregation.

### Production of a Recombinant Enzyme Is Increased in the AG-GAGΔ-cutL1 Strain

**Figure 9:**
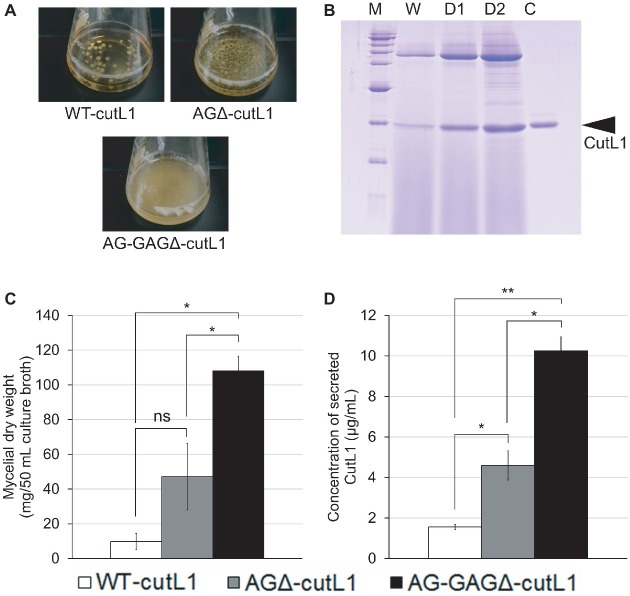
Recombinant CutL1 production by the WT-cutL1, AGΔ-cutL1, and AG-GAGΔ-cutL1 strains in liquid culture. **(A)** Phenotypes of the WT-cutL1, AGΔ-cutL1, and AG-GAGΔ-cutL1 strains under liquid culture conditions. Conidia (final concentration, 1 × 10^4^/ml) of each strain were inoculated into YPM medium and rotated at 100 rpm at 30°C for 24 h. **(B)** Secreted protein profiles of each strain. Lanes W, D1, D2: proteins precipitated from culture supernatants (250 μl) of the WT-cutL1, AGΔ-cutL1, and AG-GAGΔ-cutL1 strains, respectively; lane C: 1 μg of purified CutL1. **(C)** Mycelial dry weight of each strain. Mycelia grown for 24 h were collected by filtration through Miracloth, dried at 70°C and weighed. **(D)** Concentration of secreted CutL1 in culture supernatants. In **(C)** and **(D)**, error bars represent the standard error of the mean calculated from three replicates (*p < 0.05; **p < 0.01). ns, not significant.

We investigated whether hyphal dispersion would increase biomass and enzyme production in *A. oryzae*. As expected, hyphae of the AG-GAGΔ-cutL1 strain cultured in YPM medium were fully dispersed and those of the AGΔ-cutL1 strain formed smaller pellets than those of the WT-cutL1 strain (Figure 9A). After 24 h of culture, the culture supernatant of each strain was subjected to SDS-PAGE, resulting in greater secreted protein profiles in AG-GAGΔ-cutL1 than in the WT-cutL1 and AGΔ-cutL1 strains (Figure 9B). Both biomass and cutinase production was higher in the AG-GAGΔ-cutL1 strain (approximately 10 times) and in the AGΔ-cutL1 strain (4 times) than in the wild-type (Figures 9C,D). This result suggests that hyphal dispersion caused by a loss of the hyphal aggregation factors α-1,3-glucan and GAG can increase biomass and recombinant enzyme production in filamentous fungi that have α-1,3-glucan or GAG or both.

## Discussion

Hyphae of filamentous fungi generally form large aggregated pellets in liquid culture, thus limiting the fermentative production of commercially valuable enzymes and metabolites (Driouch et al., 2010, 2012). Aggregation of hyphae seems to be related to their cell surface properties (Fontaine et al., 2010; Priegnitz et al., 2012; Yoshimi et al., 2017), but the mechanism of hyphal aggregation is not well understood. We previously demonstrated that α-1,3-glucan in the cell wall has a role in hyphal adhesion in *A. nidulans* (Yoshimi et al., 2013; Miyazawa et al., 2018), and that the hyphae of α-1,3-glucan-deficient mutants of *A. oryzae* form smaller pellets than those of the wild-type but are not dispersed (Miyazawa et al., 2016). We concluded that α-1,3-glucan is an adhesive factor for *A. oryzae* hyphae, but another factor involved in hyphal adhesion remains in the AGΔ strain. Here, we focused on GAG, a component of the extracellular matrix, as a candidate adhesive factor. Lee et al. (Lee et al., 2016) revealed that GAG biosynthesis is controlled by five clustered genes (*gtb3*, *agd3*, *ega3*, *sph3*, and *uge3*) in *A. fumigatus*, and that similar gene clusters are conserved in various fungi, such as *A. niger* and *A. nidulans*. We found that the gene cluster is also conserved in the genome of *A*. *oryzae*.

α-1,3-Glucan contributes to hyphal and mycelial adhesion in *A. nidulans*, *A. oryzae*, and *A. fumigatus* (Fontaine et al., 2010; Yoshimi et al., 2013; Miyazawa et al., 2016). GAG mediates hyphal adhesion to plastic, fibronectin, and epithetical cells, and its function is related to pathogenesis in *A. fumigatus* (Gravelat et al., 2013). GAG also mediates biofilm formation in plate cultures (Gravelat et al., 2013). However, neither the relationship between GAG and hyphal aggregation nor the phenotype of an AG-GAG double mutant (AG-GAGΔ) has been reported. Here, we constructed AG-GAGΔ and a single mutant (GAGΔ) in *A*. *oryzae* and analyzed their growth in liquid culture. The AG-GAGΔ hyphae were completely dispersed, but the GAGΔ hyphae formed pellets larger than those of the wild-type strain (Figure 2). These results suggest that not only α-1,3-glucan but also GAG contributes to hyphal aggregation in *A*. *oryzae*.

We investigated whether α-1,3-glucan and GAG showed temporal and spatial differences in their effects on hyphal aggregation during germination and hyphal growth. Because the germ tubes of the wild-type and GAGΔ strains aggregated at 3 h after inoculation of their conidia, whereas those of AGΔ did so at 6 h, we conclude that α-1,3-glucan is present on the surface of most hyphae just after germination and acts as an adhesive factor, whereas GAG, which is secreted and presented around the hyphal tips, contributed to hyphal aggregation at 6 h after inoculation (Figures 4, 5). We succeeded in *in vitro* aggregation of AG-GAGΔ hyphae by adding GAG partially purified from AGΔ strains (Figure 6). In the presence of GAG, AG-GAGΔ mycelia aggregated at pH 6 and 7, but aggregation was reduced at acidic pH (Figure 7B).

In the GAG biosynthetic gene cluster, *agd3* encodes *N*-acetylgalactosamine deacetylase, and GalNAc molecules in GAG chains from *A. fumigatus* are partly deacetylated (Fontaine et al., 2011). Disruption of *agd3* in *A. fumigatus* abolishes GAG deacetylation and results in a loss of cell wall-associated GAG (Lee et al., 2016). Positively charged amino groups in deacetylated GalNAc in GAG are thought to be required for the attachment of hyphae to negatively charged surfaces (Lee et al., 2016) and likely prevent hyphal aggregation at acidic pH because of electric repulsion; these groups would be unprotonated at pH close to neutral, in particular at the putative isoelectric point of GAG. Therefore, attachment of GAG in this pH range might be attributable to hydrogen bonding between the amino groups of GalN and the OH groups of the sugar moieties in the glucan of the hyphal cell wall or GAG chains pre-attached to the cell wall. The DD of GAG derived from AGΔ was about 50% (Table 3). Addition of GAG with amino groups acetylated by acetic anhydrate barely induced mycelial aggregation (Figure 8A), and the DD of acetylated GAG of AGΔ was significantly decreased by the acetylation (Table 3). These results strongly suggest that deacetylation of GalNAc residues in the GAG molecule is important for GAG-dependent aggregation. In addition, GAG-induced mycelial aggregation was inhibited in the presence of 8 M urea (Figure 8B). These observations indicate that amino group acetylation abolishes hydrogen bonding between GAG and hyphal glucans or GAG pre-attached to hyphae. Hydrogen bonds might be a major force in GAG-dependent hyphal aggregation at pH close to neutral. When hyphae aggregated by addition of GAG at neutral pH were subsequently transferred to acidic buffer (pH 4), they remained aggregated (data not shown), suggesting that, once formed, the adhesion among GAG chains is resistant to acidic conditions.

Formation of hyphal pellets limits productivity in the fermentation industry that uses filamentous fungi, including *Aspergillus* species, because the inner part of the pellet is inactive (Driouch et al., 2010). In *A. niger*, titanate particles are used as a scaffold for hyphal pellets to minimize their size (Driouch et al., 2012). Although physical approaches are efficient, they limit the range of culture media. The AG-GAGΔ strain produced significantly larger amounts of biomass and cutinase than did the AGΔ and wild-type strains; it did not require any scaffold particles, suggesting that controlling the hyphal aggregation factors of hyphae is an innovative approach for the fermentation industry.

We demonstrated that both α-1,3-glucan and GAG on the hyphal surface contribute to the formation of hyphal pellets and are adhesive molecules. The physicochemical properties of the two polysaccharides differ. α-1,3-Glucan is a water-insoluble major cell wall polysaccharide, whereas GAG is secreted and is a water-soluble component of the extracellular matrix. Further studies are necessary to understand the molecular mechanism underlying the interactions among α-1,3-glucan and GAG chains.

## Data Availability

The datasets generated for this study are available on request to the corresponding author.

## Author Contributions

KM, AY, and KA conceived and designed the experiments. AY determined the sensitivity to LE and CR. KM and MS constructed fungal mutants. FT performed the assay of CutL1 production. KM and AS performed the Southern blot analysis. SK performed the ^13^C NMR analysis. AK and SY produced AGBD-GFP. KM and TN performed fractional precipitation of GAG. KM performed most experiments and analyzed the data.

### Conflict of Interest Statement

The authors declare that the research was conducted in the absence of any commercial or financial relationships that could be construed as a potential conflict of interest.

## Footnotes

^1^https://blast.ncbi.nlm.nih.gov/Blast.cgi

## References

[ref1] Abe K. Gomi K. Hasegawa F. Machida M. (2006). Impact of *Aspergillus oryzae* genomics on industrial production of metabolites. Mycopathologia 162, 143–153. 10.1007/s11046-006-0049-2, PMID: 16944282

[ref2] Bamford N. C. Snarr B. D. Gravelat F. N. Little D. J. Lee M. J. Zacharias C. A.. (2015). Sph3 is a glycoside hydrolase required for the biosynthesis of galactosaminogalactan in *Aspergillus fumigatus*. J. Biol. Chem. 290, 27438–27450. 10.1074/jbc.M115.679050, PMID: 26342082 PMC4645995

[ref3] Beauvais A. Fontaine T. Aimanianda V. Latgé J. P. (2014). *Aspergillus* cell wall and biofilm. Mycopathologia 178, 371–377. 10.1007/s11046-014-9766-0, PMID: 24947169

[ref4] Driouch H. Hänsch R. Wucherpfennig T. Krull R. Wittmann C. (2012). Improved enzyme production by bio-pellets of *Aspergillus niger*: targeted morphology engineering using titanate microparticles. Biotechnol. Bioeng. 109, 462–471. 10.1002/bit.23313, PMID: 21887774

[ref5] Driouch H. Sommer B. Wittmann C. (2010). Morphology engineering of *Aspergillus niger* for improved enzyme production. Biotechnol. Bioeng. 105, 1058–1068. 10.1002/bit.22614, PMID: 19953678

[ref6] Fontaine T. Beauvais A. Loussert C. Thevenard B. Fulgsang C. C. Ohno N.. (2010). Cell wall α1-3glucans induce the aggregation of germinating conidia of *Aspergillus fumigatus*. Fungal Genet. Biol. 47, 707–712. 10.1016/j.fgb.2010.04.006, PMID: 20447463

[ref7] Fontaine T. Delangle A. Simenel C. Coddeville B. van Vliet S. J. van Kooyk Y.. (2011). Galactosaminogalactan, a new immunosuppressive polysaccharide of *Aspergillus fumigatus*. PLoS Pathog. 7:e1002372. 10.1371/journal.ppat.1002372, PMID: 22102815 PMC3213105

[ref8] Gravelat F. N. Beauvais A. Liu H. Lee M. J. Snarr B. D. Chen D.. (2013). *Aspergillus* galactosaminogalactan mediates adherence to host constituents and conceals hyphal β-glucan from the immune system. PLoS Pathog. 9:e1003575. 10.1371/journal.ppat.1003575, PMID: 23990787 PMC3749958

[ref9] Gravelat F. N. Ejzykowicz D. E. Chiang L. Y. Chabot J. C. Urb M. Macdonald K. D.. (2010). *Aspergillus fumigatus* MedA governs adherence, host cell interactions and virulence. Cell. Microbiol. 12, 473–488. 10.1111/j.1462-5822.2009.01408.x, PMID: 19889083 PMC3370655

[ref28] Hattori M. Munezane S. Kato R. Kawauchi T. (2009). Evaluation of colloidal titration with potasium poly (vinylsulfate) to determine the degree of chitosan deacetylation. Chitin and Chitosan Res. 15, 13–19.

[ref10] Johnson A. R. (1971). Improved method of hexosamine determination. Anal. Biochem. 44, 628–635. 10.1016/0003-2697(71)90252-1, PMID: 5130949

[ref11] Karahalil E. Demirel F. Evcan E. Germec M. (2017). Microparticle-enhanced polygalacturonase production by wild type *Aspergillus sojae*. BioTechniques 7:361. 10.1007/s13205-017-1004-2. PMC5626666 28979834

[ref12] Kobayashi T. Abe K. Asai K. Gomi K. Juvvadi P. R. Kato M. (2007). Genomics of *Aspergillus oryzae*. Biosci. Biotechnol. Biochem. 71, 646–670. 10.1271/bbb.60550 17341818

[ref13] Latgé J.-P. (2010). Tasting the fungal cell wall. Cell. Microbiol. 12, 863–872. 10.1111/j.1462-5822.2010.01474.x, PMID: 20482553

[ref14] Lee M. J. Geller A. M. Bamford N. C. Liu H. Gravelat F. N. Snarr B. D. (2016). Deacetylation of fungal exopolysaccharide mediates adhesion and biofilm formation. mBio 7, 1–14. 10.1128/mBio.00252-16 PMC4817252 27048799

[ref15] Lee M. J. Gravelat F. N. Cerone R. P. Baptista S. D. Campoli P. V. Choe S. I. (2014). Overlapping and distinct roles of *Aspergillus fumigatus* UDP-glucose 4-epimerases in galactose metabolism and the synthesis of galactose-containing cell wall polysaccharides. J. Biol. Chem. 289, 1243–1256. 10.1074/jbc.M113.522516 24257745 PMC3894311

[ref16] Lee M. J. Liu H. Barker B. M. Snarr B. D. Gravelat F. N. Al Abdallah Q.. (2015). The fungal exopolysaccharide galactosaminogalactan mediates virulence by enhancing resistance to neutrophil extracellular traps. PLoS Pathog. 11:e1005187. 10.1371/journal.ppat.1005187, PMID: 26492565 PMC4619649

[ref17] Lee M. J. Sheppard D. C. (2016). Recent advances in the understanding of the *Aspergillus fumigatus* cell wall. J. Microbiol. 54, 232–242. 10.1007/s12275-016-6045-4, PMID: 26920883

[ref18] Maeda H. Yamagata Y. Abe K. Hasegawa F. Machida M. Ishioka R.. (2005). Purification and characterization of a biodegradable plastic-degrading enzyme from *Aspergillus oryzae*. Appl. Microbiol. Biotechnol. 67, 778–788. 10.1007/s00253-004-1853-6, PMID: 15968570

[ref19] Miyazawa K. Yoshimi A. Kasahara S. Sugahara A. Koizumi A. Yano S.. (2018). Molecular mass and localization of α-1,3-glucan in cell wall control the degree of hyphal aggregation in liquid culture of *Aspergillus nidulans*. Front. Microbiol. 9:2623. 10.3389/fmicb.2018.02623, PMID: 30459735 PMC6232457

[ref20] Miyazawa K. Yoshimi A. Zhang S. Sano M. Nakayama M. Gomi K.. (2016). Increased enzyme production under liquid culture conditions in the industrial fungus *Aspergillus oryzae* by disruption of the genes encoding cell wall α-1,3-glucan synthase. Biosci. Biotechnol. Biochem. 80, 1853–1863. 10.1080/09168451.2016.1209968, PMID: 27442340

[ref21] Mizutani O. Kudo Y. Saito A. Matsuura T. Inoue H. Abe K.. (2008). A defect of LigD (human Lig4 homolog) for nonhomologous end joining significantly improves efficiency of gene-targeting in *Aspergillus oryzae*. Fungal Genet. Biol. 45, 878–889. 10.1016/j.fgb.2007.12.010, PMID: 18282727

[ref22] Priegnitz B. E. Wargenau A. Brandt U. Rohde M. Dietrich S. Kwade A.. (2012). The role of initial spore adhesion in pellet and biofilm formation in *Aspergillus niger*. Fungal Genet. Biol. 49, 30–38. 10.1016/j.fgb.2011.12.002, PMID: 22178638

[ref23] Senju R. (ed.) (1969). ‘“Koroido tekiteiki no tyousei to hyoutei’ (Preparation and standardization of colloid titrant)’’ in “Koroido tekitei-ho” (Colloidal titration). Tokyo: Nanko-do, 39–41.

[ref24] Sheppard D. C. Howell P. L. (2016). Biofilm exopolysaccharides of pathogenic fungi: lessons from bacteria. J. Biol. Chem. 291, 12529–12537. 10.1074/jbc.R116.720995, PMID: 27129222 PMC4933471

[ref25] Speth C. Rambach G. Lass-flörl C. Howell P. L. Sheppard D. C. Speth C. (2019). Galactosaminogalactan (GAG) and its multiple roles in Aspergillus pathogenesis. Virulence, 1–8. 10.1080/21505594.2019.1568174 [Epub ahead of print]. PMC8647848 30667338

[ref26] Suyotha W. Yano S. Takagi K. Rattanakit-Chandet N. Tachiki T. Wakayama M. (2013). Domain structure and function of α-1,3-glucanase from *Bacillus circulans* KA-304, an enzyme essential for degrading basidiomycete cell walls. Biosci. Biotechnol. Biochem. 77, 639–647. 10.1271/bbb.120900 23470772

[ref27] Terayama H. (1951). Method of colloid titration (a new titration between polymer ions). J. Polym. Sci. 8, 243–253.

[ref29] Yoshimi A. Miyazawa K. Abe K. (2016). Cell wall structure and biogenesis in *Aspergillus* species. Biosci. Biotechnol. Biochem. 80, 1700–1711. 10.1080/09168451.2016.1177446, PMID: 27140698

[ref30] Yoshimi A. Miyazawa K. Abe K. (2017). Function and biosynthesis of cell wall α-1,3-glucan in fungi. J. Fungi 3:E63. 10.3390/jof3040063, PMID: 29371579 PMC5753165

[ref31] Yoshimi A. Sano M. Inaba A. Kokubun Y. Fujioka T. Mizutani O.. (2013). Functional analysis of the α-1,3-glucan synthase genes *agsA* and *agsB* in *Aspergillus nidulans*: AgsB is the major α-1,3-glucan synthase in this fungus. PLoS One 8:e54893. 10.1371/journal.pone.0054893, PMID: 23365684 PMC3554689

[ref32] Zhang S. Sato H. Ichinose S. Tanaka M. Miyazawa K. Yoshimi A. (2017). Cell wall α-1,3-glucan prevents α-amylase adsorption onto fungal cell in submerged culture of *Aspergillus oryzae*. J. Biosci. Bioeng. 124, 47–53. 10.1016/j.jbiosc.2017.02.013 28356219

