# Supplementary material for: Both Galactosaminogalactan and α-1,3-Glucan Contribute to Aggregation of Aspergillus oryzae Hyphae in Liquid Culture
Source: Front Microbiol. 2019 Sep 13;10:2090. doi: 10.3389/fmicb.2019.02090 (PMC6753227; doi:10.3389/fmicb.2019.02090)
Supplement: Supplementary file 1 [file Data_Sheet_1.docx]

Supplementary Material

**Both galactosaminogalactan and α-1,3-glucan contribute to aggregation of *Aspergillus* *oryzae* hyphae in liquid culture**

**Ken Miyazawa, Akira Yoshimi, Motoaki Sano, Fuka Tabata, Asumi Sugahara, Shin Kasahara, Ami Koizumi, Shigekazu Yano, Tasuku Nakajima, and Keietsu Abe***

*** Correspondence:** Keietsu Abe: kabe@niche.tohoku.ac.jp

**
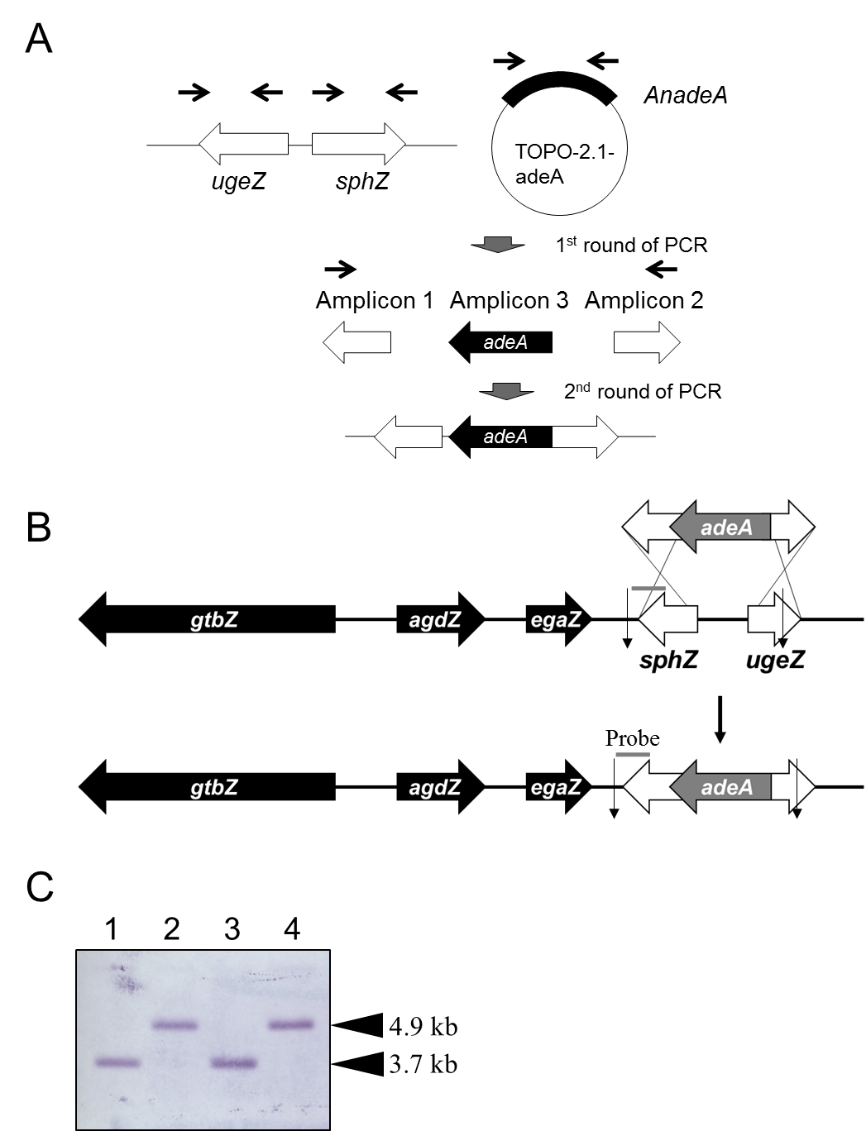
**

**FIGURE S1. Construction of *sphZ* and *ugeZ* gene disruption strains.** (**A**) Scheme of construction of the *sphZ* and *ugeZ* gene disruption cassette. (**B**) Strategy for replacement of the disrupted *sphZ* and *ugeZ* genes with the adenine requirement marker *adeA*. Thin arrows indicate *Pst*I digestion sites near the *sphZ* and *ugeZ* locus. (**C**) Southern blot analysis of the *sphZ* and *ugeZ* locus in the wild-type (lane 1), GAGΔ (lane 2), AGΔ (lane 3), and AG-GAGΔ (lane 4) strains using the probe indicated in (**B**).

**
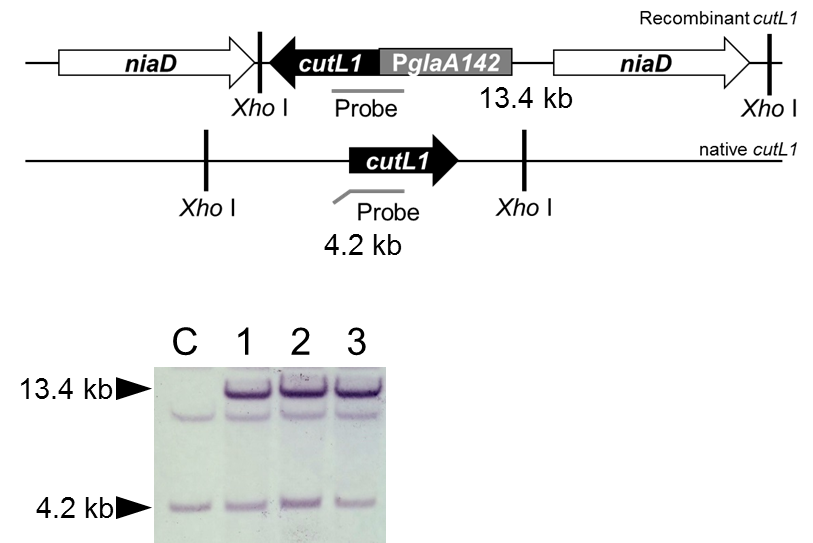
**

**FIGURE S2. Southern blot analysis of the AG-GAGΔ-cutL1 strains.** Chromosomal DNA of the control strain (lane C) and the cutL1-overexpressing strains (lanes 1–3) was digested with *Xho*I and hybridized with the probe indicated in the upper panel.

**
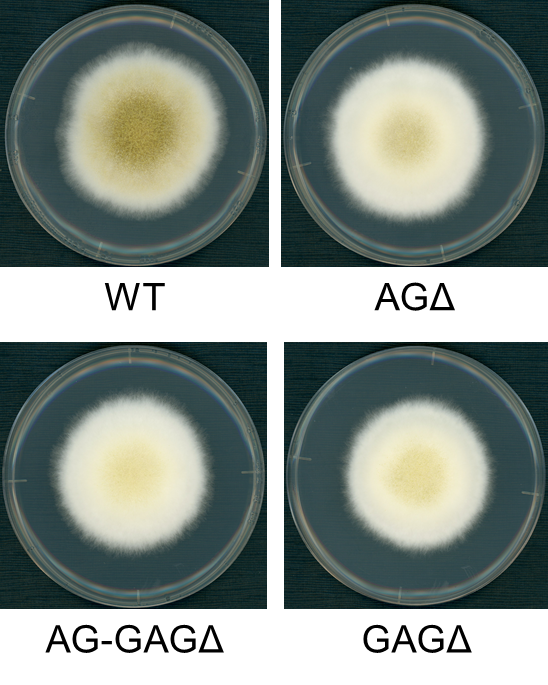
**

**FIGURE S3. Mycelial growth of the wild-type, AG**Δ**, AG-GAG**Δ**, and GAG**Δ **strains on CDE agar plates.** Conidia (1 × 10^4^) of each strain were inoculated at the center of a CDE agar plate and incubated at 30°C for 4 days


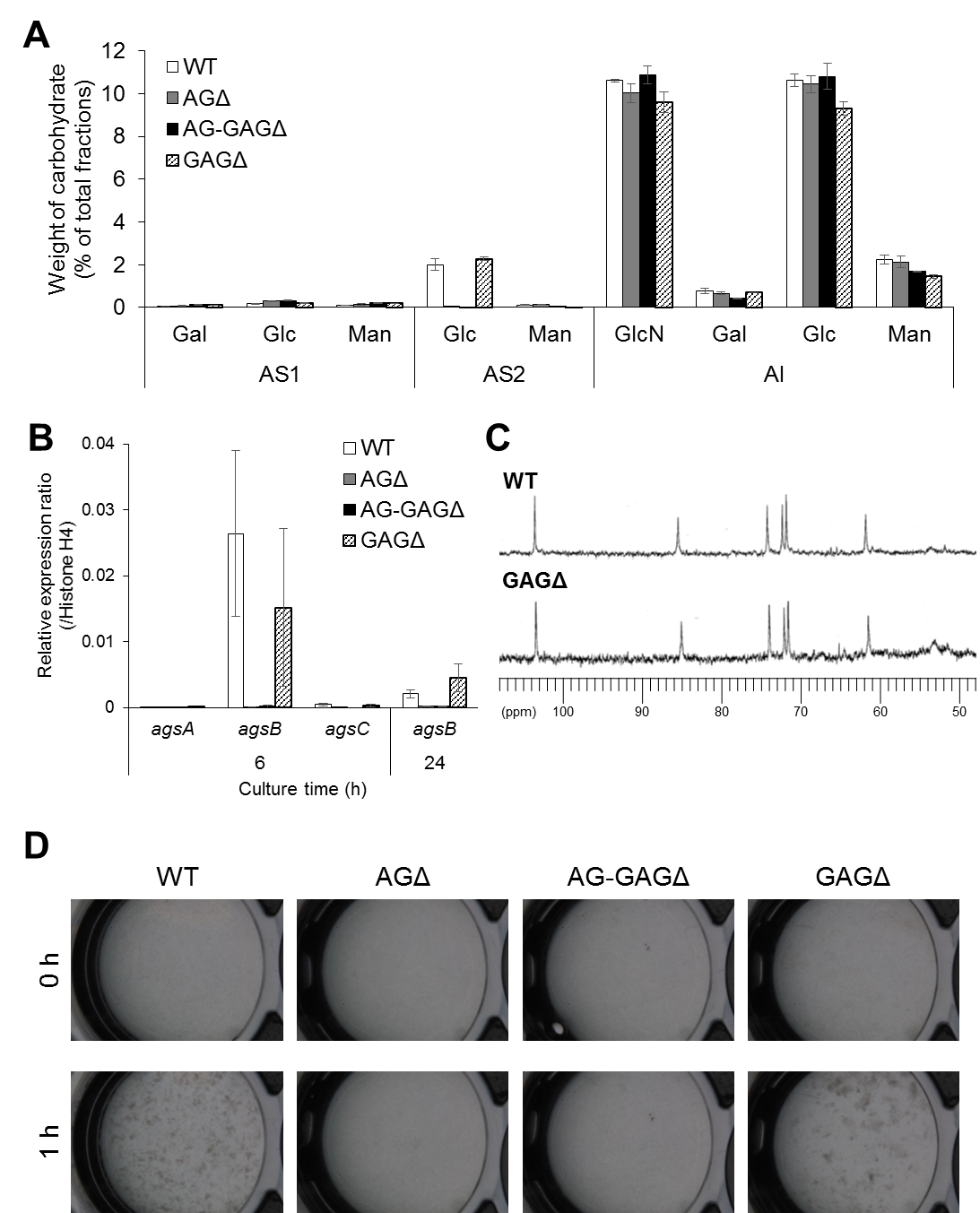


**FIGURE S4. Characterization of α-1,3-glucan in the cell wall of the wild-type, AG**Δ**, AG-GAG**Δ**, and GAG**Δ **strains.** (**A**) Monosaccharide composition of the AS1, AS2 and AI fractions. The percentage values were shown as the monosaccharide content of each fraction against the total weight of all fractions (HW + AS1 + AS2 + AI = 100%). Error bars represent the standard error of the mean calculated from three replicates. Gal, galactose; Glc, glucose; Man, mannose; GlcN, glucosamine. (**B**) Expression of the *agsA*—*C* genes in the indicating culture time. Error bars represent the standard deviation of the mean calculated from three replicates. (**C**) ^13^C NMR spectra of the AS2 fractions from the wild-type and GAGΔ strains. (**D**) Mycelial suspension of the AG-GAGΔ strain (25 µL) was added into a mixture of 400 µL of water, 50 µL of 1 M sodium phosphate buffer (pH 7.0), and 25 µL of 10 mg/mL of the AS2 fractions from the WT, AGΔ, AG-GAGΔ, or GAGΔ strains, as indicated. Samples were incubated at 30°C for 1 h with shaking and examined under a stereomicroscope (magnification, ×8).
